# The binaural masking level difference: cortical correlates persist despite severe brain stem atrophy in progressive supranuclear palsy

**DOI:** 10.1152/jn.00062.2014

**Published:** 2014-09-17

**Authors:** Laura E. Hughes, James B. Rowe, Boyd C. P. Ghosh, Robert P. Carlyon, Christopher J. Plack, Hedwig E. Gockel

**Affiliations:** ^1^Department of Clinical Neurosciences,University of Cambridge, Cambridge, United Kingdom;; ^2^Cognition and Brain Sciences Unit, Medical Research Council, Cambridge, United Kingdom;; ^3^Behavioural and Clinical Neuroscience Institute, Cambridge University, Cambridge, United Kingdom;; ^4^Wessex Neuroscience Centre, Southampton, United Kingdom; and; ^5^School of Psychological Sciences, Manchester Academic Health Science Centre, University of Manchester, Manchester, United Kingdom

**Keywords:** masking level difference, inferior colliculus, MEG, brain stem atrophy

## Abstract

Under binaural listening conditions, the detection of target signals within background masking noise is substantially improved when the interaural phase of the target differs from that of the masker. Neural correlates of this binaural masking level difference (BMLD) have been observed in the inferior colliculus and temporal cortex, but it is not known whether degeneration of the inferior colliculus would result in a reduction of the BMLD in humans. We used magnetoencephalography to examine the BMLD in 13 healthy adults and 13 patients with progressive supranuclear palsy (PSP). PSP is associated with severe atrophy of the upper brain stem, including the inferior colliculus, confirmed by voxel-based morphometry of structural MRI. Stimuli comprised in-phase sinusoidal tones presented to both ears at three levels (high, medium, and low) masked by in-phase noise, which rendered the low-level tone inaudible. Critically, the BMLD was measured using a low-level tone presented in opposite phase across ears, making it audible against the noise. The cortical waveforms from bilateral auditory sources revealed significantly larger N1m peaks for the out-of-phase low-level tone compared with the in-phase low-level tone, for both groups, indicating preservation of early cortical correlates of the BMLD in PSP. In PSP a significant delay was observed in the onset of the N1m deflection and the amplitude of the P2m was reduced, but these differences were not restricted to the BMLD condition. The results demonstrate that although PSP causes subtle auditory deficits, binaural processing can survive the presence of significant damage to the upper brain stem.

when listening to sounds in a noisy environment, subtle differences in phase and correlation between ears can greatly enhance perception. Loss of the ability to process these differences may contribute to ecologically relevant hearing loss that is not revealed by standard pure tone audiometry or otoacoustic emissions. For example, an important cue that normal-hearing listeners exploit is the slight decorrelation in waveforms that occurs when a target sound has a different location to the noise (Licklider 1948). A robust psychophysical phenomenon that reflects this critical phase processing is the binaural masking level difference (BMLD). When an identical tone is presented to each ear in the presence of masking noise that is identical in the two ears, the detection threshold can be substantially reduced, by as much as 15 dB, by inverting the polarity of the signal at one ear.

Single-cell recordings from a number of animal species have shown that the initial interaural processing important for the BMLD takes place in the medial superior olive and that an accurate representation of this processing is preserved at the level of the inferior colliculus (IC) (Asadollahi et al. 2010; Palmer and Shackleton 2002; Shackleton and Palmer 2010). Indeed, it has proven possible to provide a quantitative account of the BMLD based solely on changes in the firing rates in cells of the central nucleus of the IC (Palmer and Shackleton 2002).

The present study investigates the extent to which the preservation of that information in higher auditory structures depends on the integrity of the IC. Given that the vast majority of ascending auditory fibers from medial superior olive to thalamus are relayed through the IC, any damage to the connections might be expected to result in a reduction in cortical potentials. A physiological correlate of the BMLD was obtained from a group of patients with progressive supranuclear palsy (PSP; Richardson's syndrome), who have substantial bilateral damage to the IC, and a group of age-matched controls.

PSP is associated with progressive severe atrophy of grey and white matter in the upper brain stem, including the IC, superior cerebellar peduncle, and neighboring pathways (Josephs et al. 2008; Price et al. 2004), with more extensive degeneration in white matter tracts (Whitwell et al. 2012), and reduced midbrain volume (Massey et al. 2013). The midbrain atrophy is so prominent that it is a common MRI diagnostic marker known as the “hummingbird” or “penguin” sign, in reference to the shape of midbrain in PSP when viewed in a midsagittal plane (Groschel et al. 2006; Kato et al. 2003). Atrophic cortical regions include medial and superior prefrontal cortex, insula, frontal operculum, cingulate cortex, precentral gyrus, and superior parietal lobule (Brenneis et al. 2004; Chiu et al. 2012; Ghosh et al. 2012; Nicoletti et al. 2008). At the microscopic level the pathology includes neuronal loss, gliosis, tau-positive inclusions in both astrocytes and oligodendrocytes, and neurofibrillary tangles, especially in the brain stem, basal ganglia, and diencephalon (Dickson et al. 2007), with a greater tau burden directly affecting the IC, superior colliculus, substantia nigra, and pedunculopontine nucleus (Dugger et al. 2011). The most salient clinical manifestations of PSP are motoric. Over time, patients become severely akinetic and rigid, with loss of balance and falls, postural instability, and a vertical supranuclear gaze palsy (Litvan et al. 1996; Richardson et al. 1963). In addition to these deficits, patients also have significant cognitive changes including dysexecutive function and social impairments (Burrell et al. 2014; Ghosh et al. 2009, 2012).

Despite the bilateral damage to the IC and surrounding connections, and considering the critical position of this nucleus in the ascending auditory pathways, there have, perhaps surprisingly, been no reports of auditory deficits associated with PSP. However, this may be simply because the more debilitating aspects of the disease have led to auditory evaluations of PSP patients being restricted to simple audiometry. As noted above, the processing of interaural decorrelation is preserved at the level of the healthy IC, and so the BMLD is a prime candidate for a suprathreshold effect of damage to that region. For this patient group, a cortical neurophysiological correlate is preferable to a behavioral measure, because of the extreme bradyphrenia and motor deficits that accompany PSP. Behavioral responses are confounded by long latencies and increased risk of fatigue.

We therefore measured the cortical neural correlates of the BMLD, using magnetoencephalography (MEG). Before the main experiment, we measured the BMLD psychophysically in a cohort of young listeners with no neurological or hearing problems and obtained a bilateral neural correlate using a modification of the technique described by Sasaki et al. (2005). We then applied this technique to study the BMLD in PSP patients and in age-matched older adults. If midbrain damage produces a deficit that is at least partially specific to interaural processing, we should observe a significant interaction between tone type (in vs. out of phase at the 2 ears) and subject group (patients vs. controls). Alternatively, if PSP results in a more general deficit that affects all aspects of auditory processing, the differences between the patient and control groups should be observed in all conditions. Finally, of course, if auditory processing remains intact despite bilateral damage to the IC, no differences should be observed between the two groups.

## MATERIALS AND METHODS

### Subjects

Fourteen patients with PSP (mean age: 68; range: 58–83; 4 female) were recruited from the specialist clinic at Addenbrooke's Hospital, Cambridge, UK. The patients were diagnosed according to clinical criteria (Litvan et al. 1996) and underwent neuropsychological assessment including the following: the revised Addenbrooke's cognitive examination (ACE-r) (Mioshi et al. 2006), the mini mental state examination (MMSE) (Folstein et al. 1975), the motor section of the Unified Parkinson Disease Rating Scale (UPDRS) (Fahn and Elton 1987), and the PSP Rating Scale (PSPRS) (Golbe and Ohman-Strickland 2007). Post mortem neuropathological examination was made in six cases: all had confirmed PSP. Fifteen healthy aged-matched older adults (mean age: 70: range: 59–85; 4 female) were recruited from the volunteer panel of the MRC Cognition and Brain Sciences Unit. No subjects in the control group had a history of significant neurological, rheumatological, or psychiatric illness nor had any cognitive symptoms. The study was approved by the local Research Ethics Committee, and participants gave written informed consent.

Hearing thresholds for the subjects were measured in quiet at 500, 1,000, and 1,500 Hz using either an audiometer or a calibrated laptop that implemented the audiometric procedure. Hearing thresholds were not obtained from two patients. Two control subjects and one patient were excluded from the analyses because the medium level tone (55.5 dB SPL) was <20 dB above their 500-Hz threshold in quiet in one or both ears or because the 500-Hz quiet threshold differed by >20 dB across ears. For the remaining subjects, the threshold for the 500-Hz pure tone (the frequency of the signal used for assessing the BMLD) in quiet for the better ear did not differ significantly between the control and the PSP groups [two-tailed independent-measures *t*-test: *t*_(22)_ = 1.2, *P* = 0.24]. The thresholds for higher frequencies were higher in the PSP group than in the control group [15.1 vs. 6.2 dB SPL, *t*_(11.4)_ = 3.7, *P* ≤ 0.01; and 17.0 vs. 6.2 dB SPL, *t*_(11.3)_=3.2, *P* ≤ 0.01; for 1,000 and 1,500 Hz, respectively; degrees of freedom were downwards corrected if Levene's test showed that equal variances could not be assumed for the 2 groups]. In total, 13 controls and 13 patients were included in the analyses (details are summarized in Table 1).

**Table 1.** Details of patients and healthy controls in the main experiment

	Age	Male/Female	Years Diagnosis	MMSE/30	ACE-R/100	UPDRS/108	PSP Rating Scale/100	Hearing Threshold at 500 Hz, dB SPL
Patients		9 M, 4 F						
Mean	68		4.3	26.9	82.9	29.2	29.0	14.3
SD	6.8		3.1	2.9	8.1	14.3	10.8	6.4
Controls		9 M, 4 F						
Mean	70							11.5
SD	7.4							4.1

MMSE, 30-point mini mental state examination; ACE-R, 100-point Addenbrooke's cognitive examination revised; UPDRS, Unified Parkinson's Disease Rating Scale.

Hearing threshold is based on lower threshold across the 2 ears.

### BMLD Paradigm

The paradigm used to study the BMLD response was implemented using E-prime 2.1 software (www.pstnet.com) in Windows XP (www.microsoft.com). The stimuli were as described in detail below. In brief, in the main MEG experiment, a 64-dB SPL white noise masker, low-pass filtered at 2.5 kHz, was presented in phase (identically) to both ears (N_0_) for 1,000 ms. One out of 10 different noise exemplars was drawn at random for each presentation (trial). The time delay between trials (noise onsets) was 0 ms. The target was a 500-Hz pure tone, presented for 100 ms with an onset at 450 ms after the onset of the masker. In N_0_S_0_ conditions, the target was presented in phase to both ears. Target levels were 61.2, 55.5, and 46.1 dB SPL in the high-, medium-, and low-level N_0_S_0_ conditions, respectively. The high- and medium-level N_0_S_0_ tones were audible to young adult listeners, while the low-level N_0_S_0_ tone was not. The low-level signal was also presented in opposite phase (N_0_Sπ), making it audible for young normal hearing subjects (described below). There were 210 trials for each of the 5 conditions (noise only, high-Level N_0_S_0,_ medium-level N_0_S_0_, low-level N_0_S_0_, and low-level N_0_Sπ).

### Stimuli

Two preliminary behavioral and one preliminary MEG experiment were conducted with healthy adults to determine the exact stimulus parameters to be used in the main MEG experiment. The aim of these initial experiments was to first establish the level of the tone to be used in the low-level N_0_S_0_ and N_0_Sπ conditions to maximize the BMLD for the MEG measurements and second to determine a (medium) level for the N_0_S_0_ tone that would result in a percept that was equal in loudness (salience) as the N_0_Sπ tone. These experiments are described below.

### First Preliminary Behavioral Experiment with Healthy Adults

Five normal hearing subjects (mean age: 35; range: 27–47) were tested behaviorally to determine the stimulus parameters to be used in the MEG experiments. A target pure tone (100 ms, 500 Hz) was presented in a 1-s noise masker (starting 450 ms after noise onset). The masker was presented in phase to both ears. The tone was presented either in phase across ears, or out of phase across ears, to reveal the BMLD. The BMLD was defined as the difference between the masked threshold for the tone when it was in phase (N_0_S_0_ condition) and when it was out of phase (N_0_S_π_ condition). The noise stimuli were 1-s segments of a pregenerated 10-s white noise that was low-pass filtered at 2.5 kHz (slope of 96 dB/octave) and had a root-mean-square level of 58 dB SPL after filtering. For each trial, 1 out of 10 nonoverlapping segments was chosen randomly. Masked thresholds for the tone in the noise were measured using a two-interval two-alternative forced-choice, three-down one-up adaptive procedure. At the start of each adaptive run, the level of the tone was 61.5 dB SPL and clearly audible. The tone level was reduced after three consecutive correct answers and increased after an incorrect answer. The step size was 6 dB for the first two reversals, 4 dB for the next two reversals, and 2 dB thereafter. The level at each turn point was recorded, and the last 8 from a total of 12 turn points were averaged to represent the run. Subjects completed between three and six adaptive runs for each condition, and the mean of these runs was taken as threshold. Noise stimuli had 100-ms onset and offset ramps, while tone stimuli had 10-ms onset and offset ramps; ramps were hanning shaped and included in the overall stimulus duration. Stimuli were presented via ER-3A Etymotic insert earphones with plastic tubing extended to 2 m, the same as used for the MEG experiments. The sound output of the acoustic systems (at the end of the extension tubes) was calibrated using a Type 45DA KEMAR artificial head assembly. The masked-tone thresholds averaged across listeners were 43.2 and 28.1 dB SPL for the N_0_S_0_ and the N_0_S_π_ conditions, respectively. The measured BMLD of 15.1 dB corresponded well to values reported previously for broadband maskers and low-frequency signals (Durlach and Colburn 1978; Hirsh 1948; Hirsh and Webster 1949). Based on these results, two tone levels were chosen for the preliminary MEG experiment with young healthy listeners; the noise stimuli were identical to those used in the behavioral experiment. In the high-level N_0_S_0_ condition, the 500-Hz tone was presented at 55.2 dB SPL, i.e., 12 dB above threshold, thus making it clearly audible. In the low-level N_0_S_0_ condition, the 500-Hz tone was presented at 40.1 dB SPL, i.e., 3.1 dB below the averaged threshold for this condition, rendering it inaudible for most subjects (most of the time). In contrast, the tone at the same low (40.1 dB SPL) level was audible in condition N_0_S_π_; here it was 12 dB above averaged masked threshold. Choosing this low signal level (around masked threshold in the N_0_S_0_ condition) to investigate the BMLD was expected to maximize its size both psychophysically (Townsend and Goldstein 1972) and in the neuromagnetic response (Sasaki et al. 2005).

### Preliminary MEG Experiment with Healthy Adults

A preliminary MEG experiment with 11 young healthy listeners (mean age: 26; 6 female) was used to test the auditory cortical response to the high- and low-level N_0_S_0_ conditions and to the low-level N_0_S_π_ condition. Seventy trials were presented for each condition (noise only, high-level N_0_S_0,_ low-level N_0_S_0_, and low-level N_0_Sπ), and an average of 67 trials (SD = 3.4) per condition for each subject were included in the analysis. Preprocessing and source analysis included the same procedure as described below in the main MEG experiment. Bilateral dipole models were used to examine the peak amplitudes and peak latencies of the averaged N1m for each subject. A repeated-measures ANOVA of peak amplitude compared tone type (high-level N_0_S_0_, low-level N_0_S_0_, and low-level N_0_Sπ) by dipole source (left or right auditory cortex). There was a significant difference in peak amplitude across tones [mean high-level N_0_S_0_: 19.7 nAm; low-level N_0_S_0_: 2.6 nAm; and low-level N_0_Sπ: 13.8 nAm; *F*_(2,20)_ = 33.9, *P* < 0.001]. Pairwise *t*-tests revealed that each pair was significantly different from each other (*P* < 0.05), but there was no difference in dipole source [*F*_(2,48)_ = 0.9, *P* > 0.05]. An ANOVA comparing peak latency of the high-level N_0_S_0_ and low-level N_0_Sπ tones by dipole source revealed a significant difference between tone types [mean high-level N_0_S_0_: 150 ms after tone onset; and low-level N_0_Sπ: 161 ms; *F*_(1,10)_ = 6.2, *P* < 0.05], indicating a later N1m peak for the low-level N_0_Sπ tone.

These results show that the auditory cortical response was higher for the low-level N_0_Sπ condition than for the low-level N_0_S_0_ condition, thus revealing a cortical neural correlate of the BMLD. In addition, the auditory cortical response was higher for the high-level N_0_S_0_ condition than for the low-level N_0_Sπ condition. This indicates that even though in both conditions the level of the tone was 12 dB above masked threshold (the same “sensation level”), it likely was more salient in the high-level N_0_S_0_ condition than in the low-level N_0_S_π_ condition. This interpretation is consistent with the finding that the psychophysically measured BMLD is generally smaller for suprathreshold measures such as loudness than for detection threshold (Townsend and Goldstein 1972). It is also consistent with the findings of Sasaki et al. (2005), who reported a decrease in the size of the BMLD measured in MEG (N1m amplitude) with increase in signal level relative to the psychophysically measured threshold in the N_0_S_0_ condition, in young normal hearing subjects.

### Second Preliminary Behavioral Experiment with Healthy Adults

To determine a medium-level tone that would give equal salience to the low-level N_0_S_π_ condition, a second preliminary behavioral experiment was conducted on six normal hearing subjects (mean age: 43; range: 29–58). In a two-interval task, subjects were presented first with a stimulus of the low-level N_0_S_π_ condition. The second interval contained a N_0_S_0_ stimulus with variable level of the tone in the fixed level noise; tone levels could range from 40.2 dB SPL (close to low-level) to 55.2 dB SPL (the high level) in 1 dB steps. Listeners adjusted the tone level in the second interval, so that its loudness matched the loudness of the tonal percept in the first interval. The initial tone level was chosen randomly from within the possible range of levels. Each subject made nine matches. The loudness of the tone in condition low-level N_0_S_π_ was matched with a tone of 49.5 dB SPL in condition N_0_S_0_, i.e., 9.4 dB higher than in the low-level N_0_S_0_ condition and 5.7 dB lower than that used in the high-level N_0_S_0_ condition. Therefore, in the main MEG experiment with older subjects, these three relative tone level differences were used, in which the N_0_S_0_ tone was either at the same physical level, or the same loudness, or the same sensation level as the N_0_S_π_ tone. Note that, to compensate for expected general hearing changes in the older population, all stimulus levels including that of the noise were increased by 6 dB in the main MEG experiment. Thus the noise level was 64 dB SPL, while the tone levels were 61.2, 55.5, and 46.1 dB SPL in high-, medium-, and low-level N_0_S_0_ conditions, respectively, and 46.1 dB SPL in the N_0_S_π_ condition_._

### Magnetoencephalography and Data Processing

A 306-channel Vectorview system (Elekta Neuromag, Helsinki, Finland) in a light Elekta-Neuromag magnetically shielded room was used for MEG. A magnetometer and two orthogonal planar gradiometers were located at each of 102 positions. Paired EOG electrodes measured vertical and horizontal eye movements. Head position was monitored using five head-position indicator (HPI) coils. The three-dimensional locations of the HPI coils and ∼80 “head points” across the scalp, and three anatomical fiducials (the nasion and left and right preauricular points), were recorded using a 3D digitizer (Fastrak Polhemus, Colchester, VA). Data were preprocessed using MaxFilter software (Elekta-Neuromag) with compensation for movement and down sampled from 1,000 to 500 Hz. The data were then processed using Brain Electrical Source Analysis (BESA version 5.3).

Data were high-pass filtered above 0.1 Hz (Butterworth filter 6 dB/oct with no added padding). Artifact detection and eye blink correction used Brain Electrical Source Analysis (BESA version 5.2). The artifact rejection threshold was set to 2,500 fT for magnetometers and 900 fT for gradiometers. Eye-blinks were identified and corrected using BESA's adaptive artifact correction. Epochs (−100–800 ms) were time locked to the onset of the noise, baseline corrected (−100-0 ms), and band-pass filtered (1–40 Hz). Epochs for each signal tone type were averaged and contrasted with the average of those for the noise alone. After artifact rejection, the healthy controls had an average of 178 trials each for the 5 conditions (SD = 25) and patients had 164 trials (SD = 33). The averages for each condition were used for the source analysis per subject.

#### Source analyses 1: auditory cortical waveforms.

Source analysis of gradiometer data (band-pass filtered 1–40 Hz) was used to examine the evoked response to each tone type in bilateral auditory cortex. In BESA 5.3 a realistic head model was coregistered by fiducial and digitized scalp loci and used to estimate the forward model topography (leadfield). The inverted leadfield matrix was applied to the gradiometer data to estimate two bilateral equivalent current dipoles across a 100-ms interval for the peak N1 response (150 ms after tone onset, ±50 ms). For each subject, dipole estimates were first obtained for the high-level N_0_S_0_ tone by iteratively varying the source location and orientation to minimize residual difference between scalp and model waveforms with 1% regularization constant to stabilize source fitting in the presence of noise. Fitting was constrained by symmetry on the two source's locations to ensure right and left auditory cortical sources were modeled efficiently. For the medium-level N_0_S_0_, low-level N_0_S_0_, and low-level N_0_Sπ, the subject-specific dipole location obtained for the high-level N_0_S_0_ tone was used, and only the orientation fitted iteratively. This procedure ensured that the dipoles were placed optimally for the auditory cortex for all tones, whether perceived or not.

Two components of the auditory waveforms were examined: the initial N1m and the subsequent P2m. For each component, the peak amplitude of response and latency of peak response for each dipole were entered into an ANOVA to compare responses to the medium-level N_0_S_0_, low-level N_0_S_0_, and low-level N_0_Sπ tones and between the patient and control groups. Pairwise comparisons were Bonferroni corrected for multiple comparisons and Greenhouse-Geisser corrected where appropriate. To estimate differences between the source waveform contours, two-sample *t*-tests were calculated time point by time point for each condition between patients and controls across the 100- to 300-ms time window after tone onset for the N1m and P2m. Differences were considered significant if data points consecutively met the *P* < 0.05 criterion for at least 22 ms (11 data points with 500-Hz sampling). This method is considered a suitable, more liberal alternative to using the Bonferroni method (cf. Guthrie and Buchwald 1991).

#### Source analyses 2: distributed source analysis.

A second model examined the distributed cortical response, using standardized low-resolution brain electromagnetic tomography (sLORETA; Pascual-Marqui 2002), a type of distributed source analysis that computes a current distribution across the entire brain volume. sLORETA images were computed for each subject and each tone type across two time windows: the N1 time window (100 to 200 ms after tone onset) and the P2 time window (200–300 ms after tone onset). To compare group differences, statistical cluster-based permutation comparisons of patients and controls, using a Fieldtrip toolbox for BESA (Oostenveld et al. 2011), were performed. Results are presented for gradiometer MEG channels on the averaged data for each subject group.

### Voxel-Based Morphometry

The voxel-based morphometry (VBM) included T1-weighted MPRAGE images for all the subjects. MPRAGE Images [repetition time (TR): 2,300 ms; echo time (TE): 2.86 ms; inversion time: 900 ms; flip angle: 9°; matrix dimensions: 192 × 192 in 144 slices with isotropic voxels: 1.25 mm] were acquired on a 3 Tesla Siemens MAGNETOM TrioTim Syngo MR B17 scanner (Siemens Medical Systems, Erlangen, Germany). Image preprocessing used SPM 8 (www.fil.ion.ucl.ac.uk/spm), the DARTEL toolbox (Ashburner 2007), and SUIT toolbox (http://www.icn.ucl.ac.uk/motorcontrol/imaging/suit.htm). The MPRAGE images from each subject were processed twice: once for whole brain analyses and secondly to focus on the brain stem structures.

The first analysis used the DARTEL toolbox and followed the steps suggested by Ashburner (2007). The T1 images for all subjects were segmented into grey, white, and cerebrospinal fluid tissue classes and together used to create a study-specific group template to improve intersubject alignment during normalization. The template was registered to Montreal Neurological Institute (MNI) space and used to generate Jacobian scaled modulated grey and white matter images from each subject that were spatially normalized to MNI space and smoothed with an 8-mm full width at half-maximum (FWHM) kernel. The second analysis utilized the SUIT tool box to focus on the brain stem and has the benefit of a high-resolution atlas of these structures for optimal normalization (Diedrichsen et al. 2006, 2009, 2011), in combination with the DARTEL functions for segmentation. MPRAGE images from each subject were cropped to include the brain stem and cerebellum. The segmentation of the cropped images followed the steps described above and were then spatially normalized preserving relative volumes of grey and white matter to the SUIT high-resolution atlas of the cerebellum and brain stem (Diedrichsen 2006; Diedrichsen et al. 2009, 2011). These images were smoothed with an 8 mm FWHM kernel.

Separate general linear models included the grey and white matter images for each subject from the whole brain and cropped methods to examine differences between patients and controls. For each general linear model, total intracranial volumes from each subject were included as nuisance covariates to correct for intersubject differences in global brain volume. Age was also included as a covariate. Statistical maps were thresholded with a cluster-based familywise error correction *P* < 0.05 (after *P* < 0.001 voxelwise uncorrected threshold).

## RESULTS

**Fig. 1.**
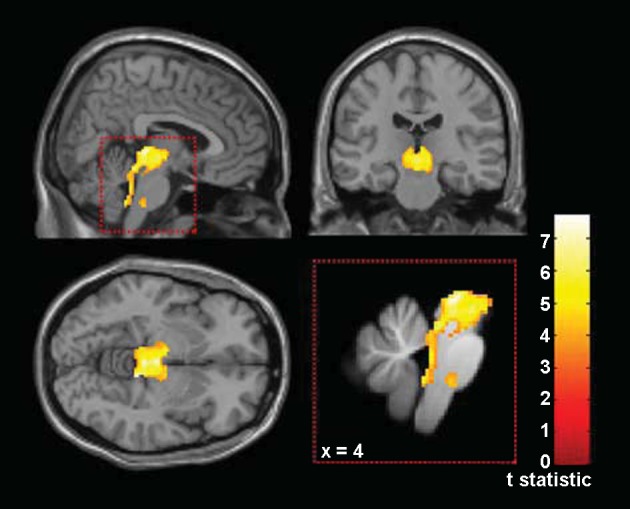
Voxel-based morphometry of white matter loss in patients compared to controls, confirmed in our group of patients to be particularly severe in the brain stem including the midbrain, cerebral peduncles, and cerebellar tracts, displayed at *P* < 0.001, unc. and overlaid on the SUIT template. Mean peak white matter loss, in the region of the inferior colliculus (IC), is 36% (SD = 14%).

The VBM of the brain stem confirmed extensive white matter atrophy for the patient group in the mid-brain and upper pons surrounding the IC, as well as the cerebral peduncles (Fig. 1). The peak white matter loss was over the IC [MNI coordinates 6 −28 −1], with ∼36% tissue loss (SD = 14%) in PSP patients compared with the healthy control group. Atrophy as measured using VBM is a late rather than early correlate of neuropathology and is indicative of further underlying pathology (such as the tau-positive inclusions and neurofibrillary tangles), which VBM cannot measure. The whole-brain VBM revealed additional regional differences in grey matter in the left anterior cingulate. There were no significant correlations between grey or white matter loss with peak amplitude or onset latency of the N1m or the P2m response to the low-level N_0_Sπ tone or the high-level N_0_S_0_ tone.

**Fig. 2.**
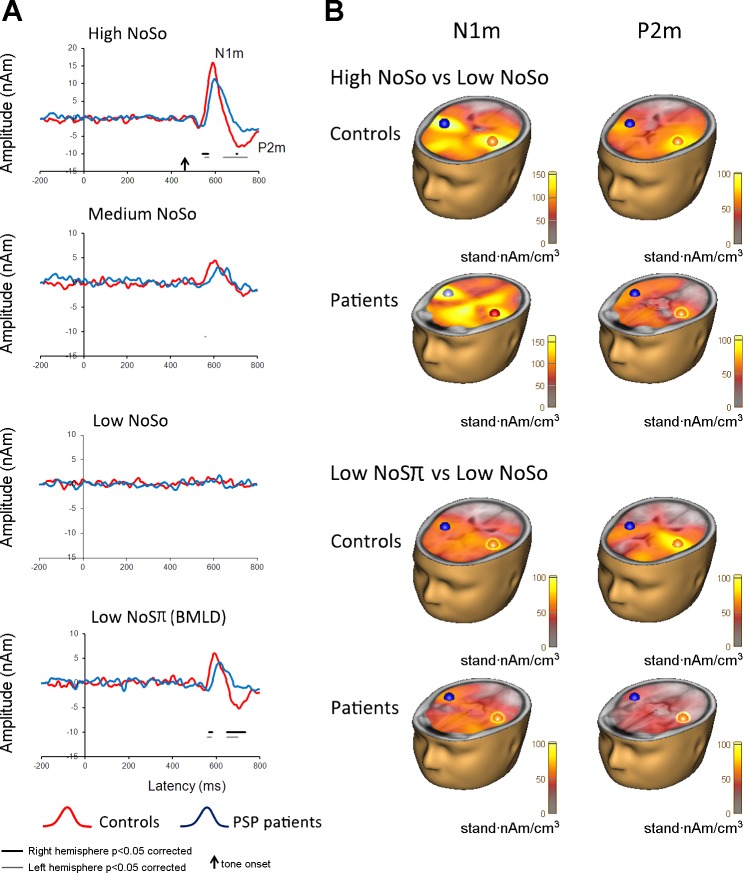
*A*: mean waveforms measured for the high-level (High) N_0_S_0_, medium-level (Medium) N_0_S_0_, low-level (Low) N_0_S_0_, and low-level (Low) N_0_Sπ tones for the patient and control groups. There is a clear N1m peak response in patients and controls for all tones except the low-level N_0_S_0_ tone. The P2m peak for the low-level N_0_Sπ tone is clear for the controls but significantly reduced in patients. Significant differences between patients and controls on a point by point basis are indicated by black and grey horizontal lines, for right and left hemisphere, respectively. *B*: sLORETA images of the N1m and P2m time window for controls and patients contrasting the high-level N_0_S_0_ vs. low-level N_0_S_0_, and low-level N_0_Sπ vs. low-level N_0_S_0_. Dipole locations are indicated with red and blue spheres.

Grand averaged source waveforms for each of the four tone types for the patient and control groups are depicted in Fig. 2*A*. For each waveform, the masking noise started at *time 0*, and tone onset was at 450 ms. Peak amplitudes and latencies are reported in Tables 2 and 3.

**Table 2.** Peak amplitudes in nAm for the N1m and the P2m

	High N_0_S_0_		Medium N_0_S_0_		Low N_0_S_0_		Low N_0_Sπ	
	Left	Right	Left	Right	Left	Right	Left	Right
N1m								
Controls	18.69 (2.2)	17.82 (1.9)	7.72 (1.1)	7.01 (0.6)	4.58 (0.6)	5.36 (0.4)	9.62 (1.2)	9.15 (0.9)
Patients	17.06 (1.8)	14.61 (2.1)	6.41 (0.9)	5.46 (1.4)	5.37 (0.7)	4.43 (0.8)	8.37 (1.3)	7.75 (1.6)
P2m								
Controls	10.69 (1.3)	13.14 (1.1)	4.75 (0.8)	6.92 (1.1)	3.24 (0.5)	4.57 (0.7)	7.51 (0.8)	9.30 (1.1)
Patients	8.25 (2.1)	6.67 (1.6)	5.43 (1.5)	3.85 (1.0)	4.16 (0.6)	3.65 (0.6)	3.97 (0.8)	5.07 (1.0)

SE is in parentheses.

N1m, 550–650 ms; P2m, 650–750 ms.

**Table 3.** Peak latency in ms for the N1m and the P2m

	High N_0_S_0_		Medium N_0_S_0_		Low N_0_Sπ	
	Left	Right	Left	Right	Left	Right
N1m						
Controls	138.62 (2.2)	142.92 (2.0)	156.77 (1.2)	158.77 (0.6)	148.92 (1.2)	153.23 (0.9)
Patients	156.31 (1.9)	159.69 (2.2)	165.85 (1.0)	167.54 (1.4)	156.31 (1.3)	155.69 (1.6)
P2m						
Controls	252.31 (7.1)	264.00 (5.9)	267.85 (8.3)	258.92 (8.5)	244.15 (6.7)	252.15 (1.1)
Patients	269.54 (8.4)	277.38 (4.5)	257.85 (8.5)	262.77 (6.6)	263.69 (9.2)	256.00 (8.7)

SE is in parentheses. N1m, 100–200 ms; P2m, 200–300 ms. NB: peak latency was not measured for the low N_0_S_0_ tone because there was no clear detectable peak.

For the high-level N_0_S_0_ tone, *t*-tests were used to examine group differences for the peak amplitudes and peak latency of the N1m and the P2m for the left and right dipole sources. We report statistics significant at *P* < 0.006 (Bonferroni corrected) and also *P* < 0.05 for transparency. There were no significant differences in peak amplitude for the N1m. Patients had a significant reduction in amplitude for the right dipole source for the P2m [*t*_(24)_ = 3.3, mean difference = 6.4 nAm, *P* = 0.003]. Trends for peak latency differences were evident for both left and right dipole sources for the N1m, revealing a delayed latency in patients [left: *t*_(24)_ = 2.8, *P* = 0.01, mean difference = 17.6 ms; right *t*_(24)_ = 2.1, *P* = 0.04, mean difference = 16.7 ms] but not for the P2m. These differences between patients and controls were confirmed in the point-by-point analysis, which revealed that patients had a significant reduction in amplitude at the N1m onset at ∼102 and 92 ms after tone onset in the left and right hemisphere, respectively. For the P2m, amplitude differences started at 188 and 246 ms in left and right hemispheres, respectively, and were sustained. These results suggest delayed onsets of both deflections and a reduced amplitude of the P2m.

For the tone types medium-level N_0_S_0_, low-level N_0_S_0_, and low-level N_0_Sπ, the peak amplitudes for the N1m and the P2m were analyzed separately using three-factor ANOVAs of tone type (medium-level N_0_S_0_, low-level N_0_S_0_, and low-level N_0_Sπ) by dipole source (left or right) by group (PSP and controls).

For the N1m, there was a significant difference in peak amplitude across tones [*F*_(2,48)_ = 14.7, *P* < 0.001], with pairwise comparisons showing significant differences between all three tone types (low-level N_0_Sπ > low-level N_0_S_0_ tone, mean difference = 3.78 nAm, *P* = .001; low-level N_0_Sπ > medium-level N_0_S_0_, mean difference = 2.0 nAm, *P* = 0.007; medium-level N_0_S_0_ > low-level N_0_S_0_ tone, mean difference = 1.7 nAm, *P* = 0.04). There were no interactions and no between-group differences [*F*_(1,24)_= 1.1, *P* = 0.3]. The fact that the N1m was greater in both groups for low-level N_0_Sπ than for the medium-level N_0_S_0_ condition (see Table 2) represents a “physiological” BMLD of at least 9.4 dB, corresponding to the level difference between the tones in the two conditions. The absence of either a group difference or an interaction between tone type and subject group means that our measurements of the N1m peak amplitude provide no evidence that the BMLD is reduced in PSP patients.

For the P2m, there was also a significant amplitude difference between the tone types [*F*_(2,48)_ = 7.8, *P* = 0.001], with pairwise comparisons showing significant differences only between the low-level N_0_Sπ and low-level N_0_S_0_ tone (mean difference = 2.55 nAm, *P* = 0.001). There was a trend towards a group difference [*F*_(1,24)_= 3.8, *P* = 0.06]. However, unlike for the N1m, there was a significant interaction between tone type and subject group [*F*_(2,48)_ = 4.7, *P* = 0.014]. This was driven by the low-level N_0_Sπ tone, for which pairwise comparisons showed controls had a significantly larger peak response than the patient group (mean difference = 3.8 nAm, *P* = 0.003). There were no significant between-group pairwise comparisons for the medium-level N_0_S_0_ tone (mean difference = 1.1 nAm, *P* > 0.05) and the low-level N_0_S_0_ (mean difference = 0.003 nAm, *P* > 0.05) tones. There was also a significant interaction of group with the location of dipole, with controls having a larger response in the right auditory cortex than the left auditory cortex, compared with the patients who had similar responses across the two hemispheres [*F*_(1,27)_ = 8.4, *P* = 0.01].

There were no significant group differences for peak latency in either time window. The point-by-point *t*-tests revealed significant differences between groups in the onset latency of the N1m (reflected by amplitude differences in the rising flank of the deflection): for the medium-level N_0_S_0_ condition starting at 108 ms, on average, in the left hemisphere only, and for the low-level N_0_Sπ condition in the right and left hemispheres (starting at 116 and 110 ms, respectively). For the P2m, only the low-level N_0_Sπ condition revealed a significant increase in onset latency (shown by a reduction in the amplitude of the onset flank) in patients compared with controls in the right and left hemispheres (starting at 200 ms for both). In the case of the P2m, the amplitude differences persisted throughout the whole deflection, due to the concomitant reduction in the peak amplitude of the P2m. There were no group differences in the low-level N_0_S_0_ condition (Fig. 2*A*).

Given that in the patient group we observed delayed onsets of the N1m in all audible tone conditions and reductions in peak amplitude of the P2m in both the high-level N_0_S_0_ condition and the low-level N_0_Sπ condition, we do not interpret this as a strong evidence for a deficit specific to the processing of interaural timing information.

sLORETA revealed strong cortical responses in auditory cortex to the high and medium level tones and the low-level N_0_Sπ condition for both patients and controls. There were no significant differences between the groups for the source estimates of the N1m and P2m in the four conditions. This suggests no differences between health and disease in generating the cortical sources for the BMLD (Fig. 2*B*).

## DISCUSSION

The results clearly demonstrated a neural correlate of the BMLD in healthy older adults with normal hearing in auditory cortex. The peak response for the out-of-phase signal (low-level N_0_Sπ) was significantly larger than for the in-phase signal of identical level (low-level N_0_S_0_) and also larger than the peak amplitude of the medium-level in-phase signal (medium-level N_0_S_0_). Surprisingly, the PSP patient group also revealed a similar pattern of results, despite significant atrophy of the midbrain.

As noted in the Introduction, single-cell recordings from animals have shown a convincing neural correlate of the BMLD at the level of the IC. Scalp-based EEG recordings from humans have revealed BMLD correlates only in later potentials associated with cortical processing. For example, Fowler and Mikami (1996) measured middle-latency potentials to N_0_S_0_ and N_0_Sπ stimuli and observed no consistent brain stem threshold differences in the responses to the stimuli, using either vertical or temporal electrode montages. In contrast, clear differences were obtained in a second experiment for the P2 component, whose latency of ∼150 ms is indicative of further cortical processing. Similarly, Wong and Stapells (2004) measured the auditory steady state response (ASSR) and observed a correlate of the BMLD only for a stimulus modulation rate of 7 or 13 Hz, where the response reflects cortical processing, but not for a modulation rate of 80 Hz where the response reflects the activation of lower brain stem neurons. Also, Ishida and Stappells (2009), using a similar method to Wong and Stappells, found no detectable BMLD for the 40-Hz ASSR. However, this does not mean that the brain stem is not important for the BMLD. Indeed, as Jiang et al. (1997) have shown, the addition of an Sπ tone to a diotic noise produces an increase in the firing rate of some binaurally driven neurons in the guinea pig IC but a reduction in others. Jiang et al. (1997; also see Palmer and Shackleton 2002) modeled the BMLD using a combination of different types of neurons whose firing rates changed in response to the tone, regardless of the direction of that change. Both types of change, increases and decreases, convey information, but it is unlikely that they would combine to produce a change that was detectable by scalp EEG. As spatial and temporal alignment of neuronal firing is required for measurable MEG and EEG signals, most likely the BMLD would only manifest in the evoked potentials generated above this level. Nevertheless, the brain stem and thalamus would be essential in maintaining and transmitting the information to higher levels of the auditory system.

One source of evidence on the importance of an intact brain stem for the preservation of the BMLD comes from lesion studies. Lynn et al. (1981) revealed that lesions to the pontomedullary region, but not lesions to the midbrain, thalamus, or cortex, reduced the size of the behavioral BMLD. Single-case studies have reported similar findings of spared BMLD with upper brain stem lesions but also indicate the presence of auditory impairments. A direct and normal measure of the BMLD was obtained in a young patient with a small hemorrhagic lesion restricted to the right IC but who was deficient at sound localization in contralesional space (Champoux et al. 2007). Another patient with a right brain stem lesion, likely including the IC and/or the lateral lemniscus, also resulted in poor localization of sounds to the contralesional side but spared the ability to benefit from spatial segregation of targets and maskers (Litovsky et al. 2002). However, it should be noted that this benefit, unlike the BMLD, does not depend exclusively on the processing of fine interaural time differences; additional factors, such as an increased signal-to-masker ratio at one ear, also contribute substantially to this type of masking release. More generally, Furst et al. (2000) studied auditory perception (although not the BMLD) in patients with brain stem lesions as a result of either stroke or multiple sclerosis. Of most relevance to the present study were a group of five patients that had lesions to the IC and/or left lateral lemniscus. All showed impaired measures of sound lateralization, including abnormal sensitivity to both interaural level and interaural time differences. Overall, the results of previous studies have revealed deficits associated with IC lesions for tasks that include, but are not limited to, the processing of fine interaural timing differences.

The present study measured an MEG correlate of the BMLD and found broadly similar results to those obtained previously with normal-hearing listeners (Chait et al. 2006; Sasaki et al. 2005). Sasaki et al. (2005) used MEG to determine the relation between psychophysically measured BMLD and the neuromagnetic BMLD as a function of signal level re. masked threshold in the N_0_S_0_ condition in young normal hearing listeners. The BMLD measured in MEG (the difference between N1m amplitude in conditions N_0_Sπ and N_0_S_0_) decreased when the signal level was increased above the psychophysically measured threshold in the N_0_S_0_ condition. The N1m peak latency of ∼140 ms in the N_0_Sπ condition, for the relative signal level closest to the one used here, is comparable to the 151-ms average peak latency in the low-level N_0_Sπ observed here in controls. We additionally show that the correlate is largely unaffected by an average 36% damage to the IC. For our patients, the N1m was larger in the low-level N_0_Sπ condition than in the medium-level N_0_S_0_ condition, in which the signal level was 9.4 dB higher; hence our patients exhibit a BMLD of at least 9.4 dB.

Although the BMLD was preserved in the patient group, their neural degeneration did result in some measurable effects in the evoked responses revealing possible auditory impairments that were not restricted to the N_0_Sπ condition. Specifically, *1*) although the N1m peak amplitude and latency did not differ between groups, the point-by-point analysis indicated that the N1m onset was delayed in the patient compared with the control group in the high-level N_0_S_0_, medium-level N_0_S_0_, and low-level N_0_Sπ conditions, and *2*) the P2m measured in the high-level N_0_S_0_ condition and the low-level N_0_Sπ condition showed delayed onsets and reduced peak amplitudes in patients. However, given that these differences were observed in both the antiphasic condition (N_0_Sπ, where signal and noise have opposite phases) and in homophasic conditions (where signal and noise have the same phase), we do not interpret this finding as evidence for impaired processing specific to BMLD in the patient group.

PSP has not been clinically associated with hearing loss, beyond its association with older age; however, the sensory effects of PSP have rarely been studied. Ghosh et al. (2012) found no effect of PSP on pure tone auditory thresholds across the 250- to 4,000-Hz range in a sample of 23 patients compared with age-matched controls. An earlier MEG study of the auditory mismatch negativity in PSP showed the N1 cortical responses to tones were well preserved despite a reduced mismatch negativity in the P2m time window (Hughes et al. 2013). The results of the current study are consistent with these findings.

The BMLD persisted in PSP patients, despite the severe bilateral atrophy of the IC. It is theoretically possible that subtle changes in the early cortical correlates of the BMLD might be identified with larger groups and that this study lacked power. However, this study was powered at B = 0.85 for detecting a large effect of group (d = 0.8) and B = 0.75 for detecting a medium effect size (d = 0.5), with alpha =0.05. Note though that our study was powerful enough to show a significant interaction between stimulus and subject group for the P2 component.

The most parsimonious explanation for our results, in the context of the previous lesions studies, is that the surviving tissue is sufficient to maintain the representation of the BMLD in the IC. The IC is the centre of the atrophy with ∼36% loss, indicative of additional pathology such as neurofibrillary plaques as well as secondary degeneration to neuronal death in the remaining tissue. Autopsy reports do not indicate that pathology is localized within a tonotopic map but is widespread across the whole IC and neighboring regions (Dickson et al. 2007; Dugger et al. 2011). Thus we suggest that the signal measured in auditory cortex survives despite the degradation of the critical auditory pathways.

### Summary

Human MEG identified a cortical neurophysiological correlate of the BMLD bilaterally at ∼150 ms, resulting from the increased audibility of the out-of-phase tones, which corresponded to an increase in level of at least 9.4 dB. There is a strong association of the IC with the BMLD in comparative animal models, but the cortical signature persists in PSP despite severe subcortical neurodegenerative pathology in the region of the IC. The delayed N1m and impaired P2m are suggestive of auditory perceptual impairments in the patients but were not specific to the BMLD.

## GRANTS

This work has been supported by the Wellcome Trust (Grants 088324 and 088263); Medical Research Council (G0700503 to B. C. P. Ghosh); Guarantors of Brain (to B. C. P. Ghosh); Raymond and Beverley Sackler Trust (to B. C. P. Ghosh); and National Institute of Health Research Cambridge Comprehensive Biomedical Research Centre including the CambridgeBrain Bank.

## DISCLOSURES

No conflicts of interest, financial or otherwise, are declared by the author(s).

## AUTHOR CONTRIBUTIONS

Author contributions: L.E.H., J.B.R., B.C.P.G., R.P.C., C.J.P., and H.E.G. conception and design of research; L.E.H., B.C.P.G., and H.E.G. performed experiments; L.E.H., R.P.C., and H.E.G. analyzed data; L.E.H., J.B.R., B.C.P.G., R.P.C., and H.E.G. interpreted results of experiments; L.E.H. prepared figures; L.E.H., J.B.R., R.P.C., and H.E.G. drafted manuscript; L.E.H., J.B.R., R.P.C., C.J.P., and H.E.G. edited and revised manuscript; L.E.H., J.B.R., B.C.P.G., R.P.C., C.J.P., and H.E.G. approved final version of manuscript.
